# Supplementary material for: The COVID HOME study research protocol: Prospective cohort study of non-hospitalised COVID-19 patients
Source: PLoS One. 2022 Nov 3;17(11):e0273599. doi: 10.1371/journal.pone.0273599 (PMC9632784; doi:10.1371/journal.pone.0273599)
Supplement: S1 File — (PDF) [file pone.0273599.s001.pdf]

P.O. Box 30 001, 9700 RB Groningen, The Netherlands

Medical Ethics Review Board

Phone +31 50 361 42 04
Fax +31 50 361 43 51
E-mail metc@umcg.nl

To:
Prof. A.W. Friedrich, MD Ph.D
Medical Microbiology
EB80

Enclosure(s) ----
Ref. M20.249765

Date	19 March 2020
METc number	METc 2020/158
Title	**UMCG COVID-19 patient follow-up study: Understanding SARS-Cov2 infection and patient clinical development.**
UMCG RR number	202000198

The Medical Ethics Review Board of the University Medical Center Groningen (METc UMCG) has discussed the above mentioned protocol and considered whether or not the research falls within the scope of the Medical Research Involving Human Subjects Act (WMO).

Based on the submitted documents the METc UMCG concludes that the above mentioned protocol is not a clinical research with human subjects as meant in the Medical Research Involving Human Subjects Act (WMO).

Therefore the METc UMCG has no task in reviewing the protocol and you do not need a WMO approval before you can start the research.

Please note that other legal Acts and/or guidelines, such as the Medical Treatment Agreement (WGBO), General Data Protection Regulation (GDPR) and codes of conduct of the FEDERA (Federation of Medical Scientific Institutions) may apply to the scientific research.

Kind regards,
on behalf of the Medical Ethics Review Board

B.A.

Prof. W.A. Kamps, MD Ph.D.
chairman

J. Davids, MSc
official secretary

cc.- a.tami@umcg.nl
b.t.f.van.der.gun01@umcg.nl

umcg
