# Supplementary material for: The COVID HOME study research protocol: Prospective cohort study of non-hospitalised COVID-19 patients
Source: PLoS One. 2022 Nov 3;17(11):e0273599. doi: 10.1371/journal.pone.0273599 (PMC9632784; doi:10.1371/journal.pone.0273599)
Supplement: S2 File — (ZIP) [file pone.0273599.s002.zip › ZonMw grant approval_10430 01 201 0001.pdf]

Nederlandse organisatie voor gezondheidsonderzoek en zorginnovatie

Laan van Nieuw Oost-Indië 334
2593 CE Den Haag
Postbus 93245
2509 AE Den Haag
Telefoon 070 349 51 11
Fax 070 349 51 00
www.zonmw.nl
info@zonmw.nl

University Medical Center Groningen
Faculty of Medical Science
De heer Prof. dr. H.W.G.M. Boddeke
Postbus 72
9700 AB GRONINGEN

Dossiernummer
50-56300-98-102
Ons kenmerk
2020/22891/ZONMW

Datum
13 juli 2020

Contactpersoon
Benien Vingerhoed-van Aken
Telfoon 070 515 03 13
COVID19@zonmw.nl

Onderwerp
Honorering subsidieaanvraag, projectnummer 10430 01 201 0001

Geachte heer Boddeke,

Op 16 juni heeft ZonMw uw subsidieaanvraag '*Prospective cohort study of non-hospitalised COVID-19 patients: determining length of isolation and patient clinical development at home (COVID-HOME study)*' ontvangen.
In deze brief vindt u ons besluit en leest wat u van ons kunt verwachten.

Besluit
ZonMw besluit tot honorering van uw subsidieaanvraag. U ontvangt een subsidie van maximaal € 429.022,- voor de duur van maximaal 11 maanden. Hiervan is € 5.000,- gereserveerd voor open access publicatie kosten.
Dit bedrag is inclusief eventueel verschuldigde btw.
.
Onderbouwing besluit
De beoordelingscommissie van het COVID-19 programma heeft positief geadviseerd over uw aanvraag. Het advies van de commissie is naar oordeel van ZonMw op zorgvuldige wijze tot stand gekomen. ZonMw heeft dit advies overgenomen en ten grondslag gelegd aan haar beslissing.

Relevantie
De commissie heeft het volgende relevantieoordeel over uw subsidieaanvraag gegeven: zeer relevant.

Kwaliteit
De commissie heeft het volgende kwaliteitsoordeel over uw subsidieaanvraag gegeven: goed**.** Het kwaliteitsoordeel is als volgt onderbouwd:

De commissie is van mening dat dit een innovatief voorstel betreft met virus shedding gekoppeld aan cytokines en besmettingsroutes. De commissie heeft wel zorgen dat studie erg breed is.

Aanvullende voorwaarden/aanbevelingen voor de uitvoer van uw project

- Wij verzoeken u een reactie op de volgende aspecten op te nemen in uw voortgangs- en eindverslagen:
  - U wordt verzocht aan te geven hoe u focus aanbrengt in het onderzoek gericht op kennis die bruikbaar is in de praktijk
  - ZonMw werkt in het kader van het COVID-19-programma met Health-RI, NFU en FMS aan het toegankelijk maken van data rondom COVID-19 om huidige en toekomstige vragen zo adequaat en snel mogelijk te kunnen beantwoorden. U zult in dat kader worden benaderd om hieraan deel te nemen
- Wij vragen u extra aandacht te hebben voor implementatie, opschaalbaarheid en borging van resultaten uit uw project. ZonMw wil de nieuwe kennis zo snel mogelijk beschikbaar maken voor het veld en de praktijk.
- U kunt, buiten reguliere rapportages om, door het programma secretariaat gevraagd worden om informatie en updates over de voortgang van uw project (mondeling, schriftelijk of als een presentatie) aan te leveren. We gaan ervan uit dat u uw medewerking daaraan verleent.
- Voor studies waarin sprake is van nog te includeren patiënten en/of patiënten-materiaal worden maandelijkse updates gevraagd, u ontvangt hiervoor een template.
- Voor uw project is mogelijk een consortium-/samenwerkingsovereenkomst nodig waarin de projectpartners vastleggen onder welke voorwaarden ze gezamenlijk het project gaan uitvoeren (zie bijlage voor specificaties). Daarnaast is mogelijk een sponsorovereenkomst of Letter of Commitment vereist. U ontvangt hiervoor een apart verzoek.
- Bij de uitvoering van uw project en het gebruik van (toekomstige) resultaten dienen de principes van Maatschappelijk Verantwoord Licentiëren te worden toegepast.
- Voor studies waarbij gebruik wordt gemaakt van serologische testen:
  - VWS biedt u de mogelijkheid om voor het serologisch testen **in uw project** kosteloos te putten uit de landelijke voorraad Wantai Total Ab kits. Voor meer informatie hierover en voor het aanvraagformulier graag per e-mail contact opnemen met Maaike van den Beld en Chantal Reusken via taskforce.serologie@rivm.nl.
    Budget dat voor deze testen was opgevoerd in de begroting kan dan niet opgevoerd worden bij de eindverantwoording, de begroting wordt hierop bijgesteld.
  - Mocht u in uw studie gebruik maken van andere serologische testen: Het is wenselijk dat de Wantai test gebruikt wordt om tussen studies serologie resultaten uitgevoerd op verschillende test platformen met elkaar te vergelijken. Voor dit doel zou een steekproef van de serologische bepalingen in uw project in parallel met de Wantai test uitgevoerd kunnen worden. Ook hiervoor is het mogelijk om kosteloos te putten uit de landelijke voorraad Wantai Total Ab kits. Voor meer informatie hierover en voor het aanvraagformulier graag per e-mail contact opnemen met Maaike van den Beld en Chantal Reusken via taskforce.serologie@rivm.nl.

Start project
Het project dient uiterlijk **5 september 2020** te beginnen. Gaat het project later van start, dan vervalt de honorering van uw aanvraag.
Hiervan kan alleen in zeer bijzondere gevallen worden afgeweken

Wettelijk kader en algemene voorwaarden
Op dit besluit is de volgende regelgeving van toepassing:

- De Algemene Wet Bestuursrecht
- Deze subsidieverlening is rechtstreeks gebaseerd op de Wet ZON
- De Algemene subsidiebepalingen ZonMw. U kunt deze nalezen op: www.zonmw.nl/subsidievoorwaarden
- Procedure voor aanvragers. U kunt deze nalezen op: https://www.zonmw.nl/nl/subsidies/hoe-werkt-subsidie-aanvragen . Bij punt 17 t/m 21 leest u wat er gedurende het project van u wordt verwacht.

Specifieke voorwaarden en verplichtingen
Bij dit besluit tot honorering gelden de subsidieverplichtingen die zijn opgenomen in Bijlage 1 – Subsidieverplichtingen, deze bijlage maakt integraal deel uit van dit besluit.

Betaling van de subsidie
De subsidie zal via voorschotbedragen aan u worden overgemaakt. De betalingen zijn afhankelijk van de ontvangst en goedkeuring van voortgangsverslagen en de eindverantwoording van uw project. U ontvangt het eerste voorschot als wij het ingevulde meldingsformulier hebben ontvangen.

Eindverantwoording
Binnen 13 weken na afloop van uw project dient u een eindverantwoording in. Dit is een inhoudelijk eindverslag en de financiële eindverantwoording. De financiële verantwoording dient een specificatie van de verschillende posten te bevatten. Na ontvangst en goedkeuring van de eindverantwoording vindt de definitieve subsidievaststelling en afrekening plaats.

Tussentijdse wijzigingen
Laat het ons weten als er tussentijds iets in uw plan of uw organisatie verandert. Verandering in de opzet, planning, begroting of organisatie kunnen gevolgen hebben voor uw subsidie. Pas na goedkeuring door ZonMw zijn eventuele wijzigingen toegestaan.

Wat verwachten wij nu van u?
ZonMw kan u een voorschot voor de eerste periode van uw project verstrekken. Hiervoor dient u:

- Binnen **4 weken** na de verzenddatum van deze brief bijgevoegd meldingsformulier ingevuld retour te sturen.
- Binnen **2 weken** na de verzenddatum van deze brief een Nederlandse publiekssamenvatting in ProjectNet in te vullen (maximaal 1000 tekens, inclusief spaties). ZonMw publiceert alle gehonoreerde projecten op haar website met een Nederlandse samenvatting in eenvoudige taal. Deze is bedoeld voor een breed geïnteresseerd publiek met verschillende achtergronden. Zie de schrijfwijzer op http://www.zonmw.nl/nl/over-zonmw/logo-huisstijl.

Dossiernummer
50-56300-98-102

Ons kenmerk
2020/22891/ZONMW

Datum
13 juli 2020

Vragen
Heeft u nog vragen? Neemt u dan gerust contact op met de medewerker die in het briefhoofd vermeld staat. Dat kan via e-mail: covid-19@zonmw.nl of via telefoonnummer: 070 515 03 13. Het oorspronkelijke nummer van uw aanvraag vervalt. Er geldt een nieuw dossiernummer zoals vermeld bovenaan deze brief. Houdt uw nieuwe dossiernummer bij de hand zodat wij u snel kunnen helpen.

Bezwaarclausule
Bent u het niet eens bent met dit besluit? U kunt tot 6 weken na de verzenddatum van deze brief bezwaar maken. Liever hebben wij dat u eerst contact met ons opneemt. Wij beantwoorden graag uw vragen over deze brief. Blijft u het oneens? U kunt een bezwaarschrift sturen naar het bestuur van ZonMw, t.a.v. Commissie Bezwaarschriften ZonMw, Postbus 93 245, 2509 AE Den Haag Meer informatie over bezwaar maken vindt u op de ZonMw website via www.zonmw.nl/signalerenklagenbezwaarmaken.
Ik feliciteer u met de honorering van uw subsidieaanvraag en wens u succes bij de uitvoering van uw project!

Met vriendelijke groet,
namens het bestuur van ZonMw,

Hannie Bonink
directeur programma’s

Bijlage(n)
Bijlage 1 - Subsidieverplichtingen
Meldingsformulier start project

Kopie
Dr. A. Tami, hoofdaanvrager en projectleider/penvoerder
