# Supplementary material for: The COVID HOME study research protocol: Prospective cohort study of non-hospitalised COVID-19 patients
Source: PLoS One. 2022 Nov 3;17(11):e0273599. doi: 10.1371/journal.pone.0273599 (PMC9632784; doi:10.1371/journal.pone.0273599)
Supplement: S2 File — (ZIP) [file pone.0273599.s002.zip › Correction of grant number.docx]

From: ZonMw Research COVID19 <COVID19@zonmw.nl>

Sent: 20 July 2020 12:12

To: Boddeke, HWGM

Cc: ZonMw Research COVID19; Tami, A (mmb)

Subject: Change project number honored ZonMw project | COVID-19 program focus area 1

Report form 10430 01 201 0023.pdf

Date 20-7-2020

File number 50-56300-98-102

Correspondence number 2020/24700/ZONMW

Dear Mr Boddeke,

Due to an administrative error on our part, your project number is changing. The new project number is 10430012010023 for your project 'Prospective cohort study of non-hospitalized COVID-19 patients: determining length of isolation and patient clinical development at home (COVID-HOME study)'.

We would like to see the new notification form, enclosed in this e-mail, completed and returned no later than August 7, 2020.

In addition, we request that you submit the Dutch public summary with the new project number via ProjectNet.

We hope to have informed you sufficiently.

Sincerely,

Ineke Huizing

Cluster Assistant

ZonMw Research COVID19_lijn 1

(present Mon-Tue-Thu-Fri)

Email: covid19@zonmw.nl

**ORIGINAL EMAIL MESSAGE:**

From: ZonMw Onderzoek COVID19 <COVID19@zonmw.nl>

Sent: 20 July 2020 12:12

To: Boddeke, HWGM

Cc: ZonMw Onderzoek COVID19; Tami, A (mmb)

Subject: Wijziging projectnummer gehonoreerd ZonMw project | COVID-19 programma aandachtsgebied 1

meldingsformulier 10430 01 201 0023.pdf

Datum 20-7-2020

Dossiernummer 50-56300-98-102

Correspondentienummer 2020/24700/ZONMW

Geachte heer Boddeke,

Wegens een administratieve fout van onze kant wijzigt uw projectnummer. Het nieuwe projectnummer is 10430012010023 voor uw project ‘Prospective cohort study of non-hospitalised COVID-19 patients: determining length of isolation and patient clinical development at home (COVID-HOME study)’.

Graag zien wij het nieuwe meldingsformulier, bijgevoegd in deze e-mail, ingevuld retour uiterlijk 7 augustus 2020.

Daarnaast verzoeken wij u om de Nederlandse publiekssamenvatting in te dienen bij het nieuwe projectnummer via ProjectNet.

Wij hopen u bij deze voldoende geïnformeerd te hebben.

Met vriendelijke groet,

Ineke Huizing

Clusterassistent

ZonMw Onderzoek COVID19_lijn 1

(aanwezig ma-di-do-vr)

E-mail: [covid19@zonmw.nl](mailto:covid19@zonmw.nl)
