# Supplementary material for: The COVID HOME study research protocol: Prospective cohort study of non-hospitalised COVID-19 patients
Source: PLoS One. 2022 Nov 3;17(11):e0273599. doi: 10.1371/journal.pone.0273599 (PMC9632784; doi:10.1371/journal.pone.0273599)
Supplement: S2 File — (ZIP) [file pone.0273599.s002.zip › Translation ZonMw grant approval_10430 01 201 0001.docx]

University Medical Center Groningen

Faculty of Medical Science

Mr. Prof. Dr. H.W.G.M. Boddeke

Box 72

9700 AB GRONINGEN

Subject: Grant application funding, project number 10430 01 201 0001

Dear Mr. Boddeke,

On June 16, ZonMw received your subsidy application "Prospective cohort study of non-hospitalized COVID-19 patients: determining length of isolation and patient clinical development at home (COVID-HOME study)". In this letter you will find our decision and read what you can expect from us.

**Decision**

ZonMw decides to honor your subsidy application. You will receive a subsidy of **a maximum of € 429,022 for a maximum period of 11 months**. Of this, € 5,000 is reserved for open access publication costs. This amount includes any VAT due.

**Substantiation decision**

The assessment committee of the COVID-19 program has given positive advice about your application. ZonMw believes that the committee's advice has been drawn up in a careful manner. ZonMw has adopted this advice and laid the foundation for its decision.

**Relevance**

The committee has made the following relevance assessment for your subsidy application: very relevant.

**Quality**

The committee has given the following quality opinion about your subsidy application: good. The quality assessment is substantiated as follows:

The Committee is of the opinion that this is an innovative proposal with virus shedding linked to cytokines and infection routes. The committee has concerns that the study is very broad.

***Additional conditions / recommendations for the implementation of your project***

- Please include a response to the following aspects in your progress and final reports:
  - You are requested to indicate how you focus on research that focuses on knowledge that can be used in practice
  - As part of the COVID-19 program, ZonMw is working with Health-RI, NFU and FMS to make data about COVID-19 accessible in order to be able to answer current and future questions as adequately and quickly as possible. You will be contacted to participate in this context
- We ask you to pay extra attention to implementation, scalability and assurance of results from your project. ZonMw wants to make the new knowledge available to the field and practice as soon as possible.
- You can, outside regular reports, be asked by the program secretariat to provide information and updates about the progress of your project (oral, written or as a presentation). We assume that you will cooperate with this.
- For studies in which patients and / or patient material that are yet to be included, monthly updates are requested. You will receive a template for this.
- Your project may require a consortium / collaboration agreement in which the project partners record the conditions under which they will jointly execute the project (see appendix for specifications). In addition, a sponsorship agreement or Letter of Commitment may be required. You will receive a separate request for this.
- The principles of Corporate Social Responsibility must be applied in the implementation of your project and the use of (future) results.
- For studies using serological tests:
  - VWS offers you the opportunity to use the national stock of Wantai Total Ab kits for serological testing in your project free of charge. For more information about this and for the application form, please contact Maaike van den Beld and Chantal Reusken by email at taskforce.serologie@rivm.nl.

The budget that was entered in the budget for these tests can then not be entered in the final statement, the budget is adjusted accordingly.

- - If you use other serological tests in your study: It is desirable that the Wantai test is used to compare serology results performed on different test platforms between studies. For this purpose, a sample of the serological determinations in your project could be performed in parallel with the Wantai test. For this, too, it is possible to draw from the national stock of Wantai Total Ab kits free of charge. For more information and for the application form, please contact Maaike van den Beld and Chantal Reusken by email at [taskforce.serologie@rivm.nl](mailto:taskforce.serologie@rivm.nl).

**Start project**

The project should start **September 5, 2020 at the latest**. If the project starts later, your application will not be honored. This can only be deviated from in very special cases.

**Legal framework and general conditions**

The following regulations apply to this decision:

- The General Administrative Law Act

- This grant is based directly on the ZON Act

- The General subsidy provisions ZonMw. You can read these at: www.zonmw.nl/subsidievoorwaarden

- Procedure for applicants. You can read these at: https://www.zonmw.nl/nl/subsidies/hoe-werkt-subsidie-aanvragen. You can read what is expected of you during the project at points 17 to 21.

**Specific conditions and obligations**

The subsidy obligations that are included in Appendix 1 - Subsidy obligations apply to this funding decision, this appendix is ​​an integral part of this decision.

**Payment of the grant**

The subsidy will be transferred to you via advance payments. Payments depend on the receipt and approval of progress reports and the final justification of your project. You will receive the first advance once we have received the completed notification form.

**Final justification**

You must submit a final statement within 13 weeks after the end of your project. This is a substantive final report and the final financial statement. The financial statements must contain a specification of the various items. After receipt and approval of the final report, the final subsidy determination and settlement will take place.

**Interim changes**

Let us know if something changes in your plan or your organization in the meantime. Changes in the design, planning, budget or organization can have consequences for your subsidy.

**What do we expect from you now?**

ZonMw can provide you with an advance for the first period of your project. For this you need:

- Return the enclosed notification form **within 4 weeks** after the date of sending this letter.
- Fill in a Dutch public summary in ProjectNet **within 2 weeks** after the date of sending this letter (maximum 1000 characters, including spaces). ZonMw publishes all awarded projects on its website with a Dutch summary in simple language. This is intended for a broadly interested audience with different backgrounds. See the style guide at <http://www.zonmw.nl/nl/over-zonmw/logo-huisstijl>.

**Questions**

Do you have any questions? Please do not hesitate to contact the employee mentioned in the letterhead. This can be done by e-mail: covid-19@zonmw.nl or by phone: 070 515 03 13. The original number of your application will be canceled. A new file number applies, as stated at the top of this letter. Keep your new file number at hand so that we can help you quickly.

**Objection clause**

Do you disagree with this decision? You can object up to 6 weeks after the sending date of this letter. We prefer that you first contact us. We are happy to answer your questions about this letter.

Do you continue to disagree? You can send a notice of objection to the board of ZonMw, with regard to the ZonMw Objections Committee, PO Box 93 245, 2509 AE The Hague.

**I congratulate you on honouring your grant application and wish you success in the execution of your project!**

Sincerely,

on behalf of the board of ZonMw,

Hannie Bonink

director of programs

Attachments)

Annex 1 - Subsidy obligations

Project start notification form

Copy

Dr. A. Tami, main applicant and project leader / lead party
